# Enabling protein-hosted organocatalytic transformations

**DOI:** 10.1039/d0ra01526a

**Published:** 2020-04-23

**Authors:** Alexander R. Nödling, Nicolò Santi, Thomas L. Williams, Yu-Hsuan Tsai, Louis Y. P. Luk

**Affiliations:** a School of Chemistry, Cardiff University, Main Building, Cardiff, CF10 3AT, UK. Email: lukly@cardiff.ac.uk

## Abstract

In this review, the development of organocatalytic artificial enzymes will be discussed. This area of protein engineering research has underlying importance, as it enhances the biocompatibility of organocatalysis for applications in chemical and synthetic biology research whilst expanding the catalytic repertoire of enzymes. The approaches towards the preparation of organocatalytic artificial enzymes, techniques used to improve their performance (selectivity and reactivity) as well as examples of their applications are presented. Challenges and opportunities are also discussed.

## Abstract 2

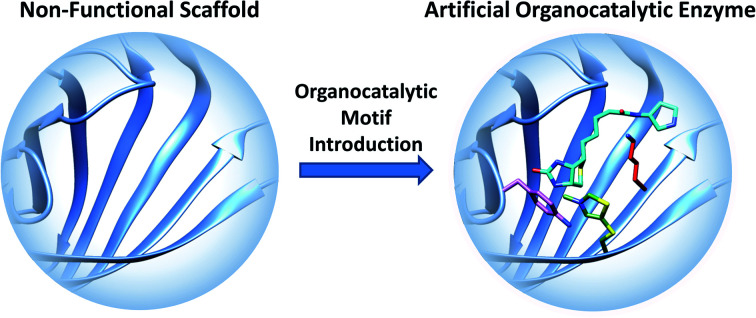
This review describes the recent approaches on integrating organocatalysis in protein systems.

## Introduction

### Biocompatible organocatalysis

Serving as a major tool for asymmetric chemical transformations,^1^–^3^ organocatalysis has now matured to a point where its bio-orthogonality can be exploited for important chemical and synthetic biology applications. Catalysts such as imidazolidinone,^4^–^6^ proline,^7^ thiourea,^8^ and anion–π^9^ derivatives have been used to mediate reactions that have no parallel in nature. Provided its bio-orthogonality, organocatalysis can be used in biological contexts for valuable chemical and biological applications.^10^–^17^ For instance, organocatalysts can serve to mediate labelling of biomolecules,^11^,^12^ analogous to existing approaches that use metals for reactions.^18^–^20^ Also, it is worth considering to merge organocatalysis and biocatalysis for the production of chiral synthons in a one-pot and atom economic fashion.^13^–^17^ However, there are only a few examples where organocatalysts function along with biomolecules or under biological conditions. In fact, taking into account the aqueous reaction medium, physiological pH (near 7.4) and temperature (near 37 °C),^20^ many of the reported organocatalysts do not function under biocompatible conditions.^8^,^10^,^17^,^21^–^31^ These boundaries vastly narrow the number of organocatalytic reactions applicable and, in response, efforts have been made to overcome limitations related to biocompatibility.^17^,^32^,^33^

### The use of proteins to host organocatalysts

To enhance the biocompatibility of organocatalysis, biomolecules including DNA, RNA and proteins can be used to host the reactions.^34^–^44^ Among them, proteins are particularly suitable. Most proteins can be made recombinantly, correctly folded in an aqueous environment under physiological conditions and are thus inherently biocompatible. Yet, the outer surfaces and interior of most proteins possess relatively low dielectric constants, which are similar to those found in many organic solvents.^45^ Consequently, proteins can provide a microenvironment that can stabilise the transition state during chemical transformation.^34^,^46^,^47^ Furthermore, superior to most organic solvents, proteins are inherently chiral – the scaffold where the catalytic motif is located can be modelled and/or genetically modified for improved selectivity.^48^ Most importantly, the protein host can be further refined by laboratory evolution, which has become increasingly facile as molecular cloning and screening techniques have become user-friendly.^49^,^50^ In contrast, it is relatively difficult to incorporate such “evolvability” in traditional catalyst design.^34^,^36^,^48^,^49^,^51^–^54^ To this end, the creation of genetically encoded protein scaffolds is a promising avenue to develop biocompatible stereoselective organocatalytic reactions.^36^,^55^

### Artificial organocatalytic enzymes

The term “artificial enzyme” has been widely used for any macromolecular complex designed to catalyse chemical reactions.^54^ Herein, we describe artificial enzymes as protein-based systems that have been genetically or chemically altered, repurposed or designed *de novo* to catalyse a reaction. While contemporary artificial enzyme design is mainly focused on metallo-enzymes or redesign of reaction-promiscuous natural enzymes,^49^,^56^ we set the scope of this review to the development of artificial organocatalytic enzymes based on recombinant proteins. These enzymes were categorised based on their design (Table 1). We will describe relevant examples of each strategy and the success in their approach. Engineering of natural cofactors in their native enzymes will be briefly discussed.^17^,^32^

**Table 1:** Advantages and disadvantages of the five approaches used for performing organocatalysis in a protein scaffold

Systems	Features	Advantages	Potential challenges	Reactions tested
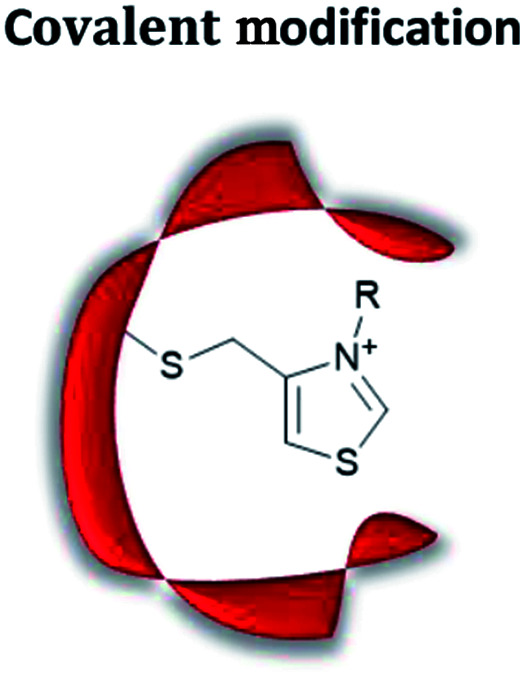	Site-selective modification with catalytically active motifs	Relative ease of preparation; quick screening of different catalytic moieties possible	Site-specific labelling can be challenging	Reduction;^57^,^58^ cyclisation;^59^ reductive amination;^60^ C–C bond formation^61^
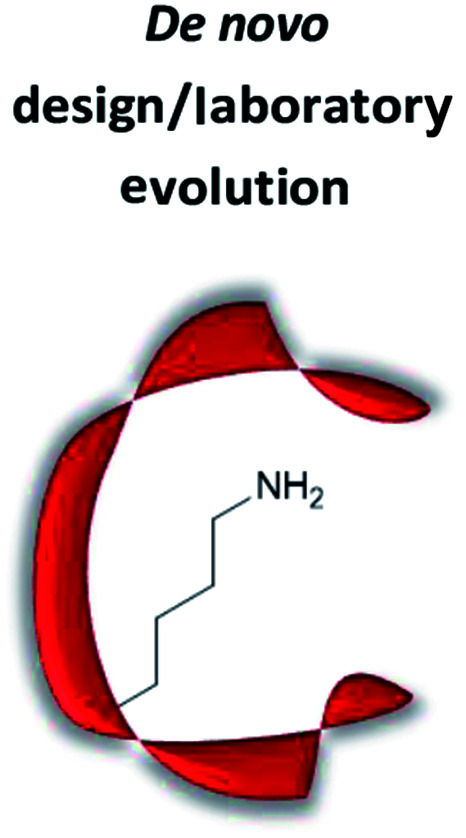	Computational design of active site, creating “theozyme”	High probability to create a novel active site as a consequence of precise design, and hence screening time is minimised	Mechanistic and structural knowledge needed; knowledge in computational chemistry needed	(Retro-)aldol reaction;^62^–^68^ Henry reaction;^41^ Knoevenagel condensation;^24^,^43^ conjugate addition^40^,^42^,^44^
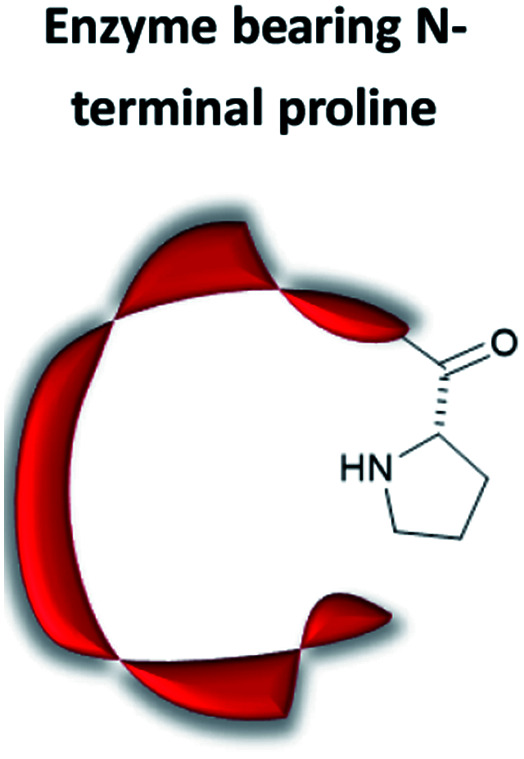	Use of substrate promiscuous N-terminal proline	No need of chemical modification or computer modelling	Limited to secondary amine organocatalysis at the N-terminal position; is it very easy to generate protein with N-terminal Pro recombinantly	Conjugate addition;^38^,^69^–^71^ intramolecular and intermolecular aldol condensation^37^,^72^,^73^
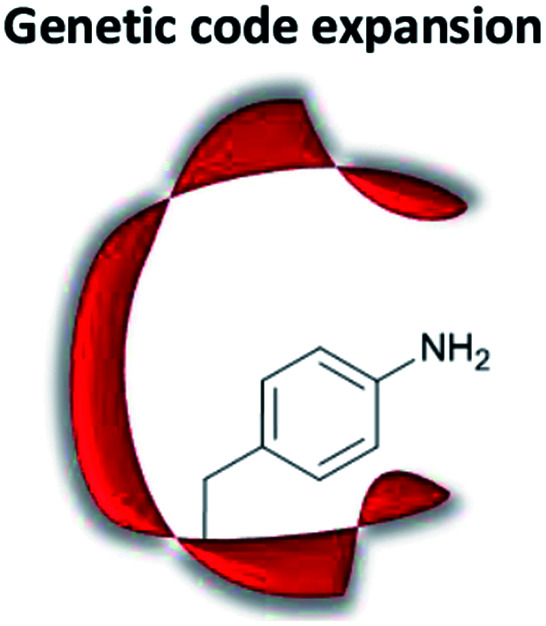	Genetic incorporation of an unnatural amino acid which bears (part of the) catalytically active motif	Wide selection of catalytically active amino acids; no chemical protein modification needed	Recombinant expression might be low yielding	Ester hydrolysis;^74^ oxime/hydrazone conjugation^50^,^75^
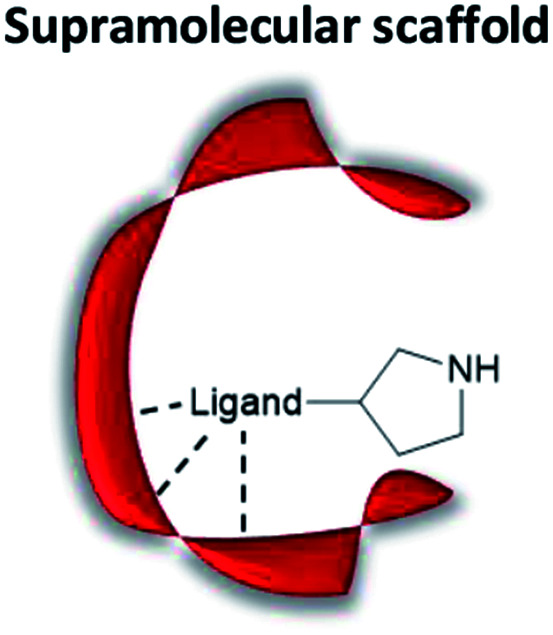	Binding of a ligand bearing catalytically active motifs	Many catalytically active moieties can be attached; enable quick screening of different catalytic moieties	Limited to proteins with high affinity ligand(s)	Conjugate addition;^39^ decarboxylative Michael addition;^36^,^55^ domino aldol–Michael reaction^76^

## Chemical modification

### Site-selective chemical modification of proteins

**Fig. 1:**
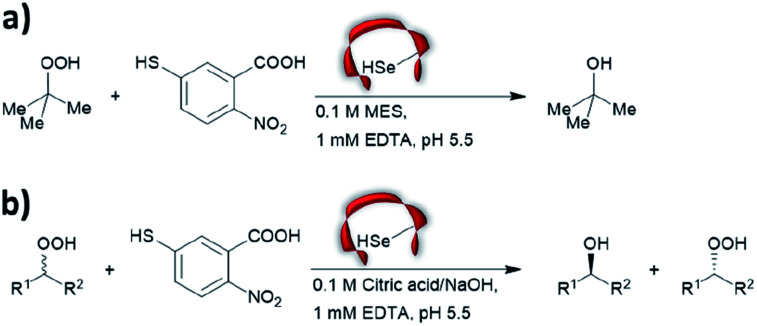
Selenosubtilisin catalyses the reduction of (a) *tert*-butyl hydroperoxide and (b) secondary alkyl hydroperoxide. MES = 2-(*N*-morpholino)ethanesulfonic acid, EDTA = ethylenediaminetetraacetic acid.

Prior to the onset of modern molecular biology technologies, proteins were often chemically modified to purposefully alter their activity.^77^–^79^ Initially, modified enzymes were made by single atom replacement. Serine protease subtilisin was converted to its cysteine equivalent by a three-step chemical protocol (tosylation, followed by replacement with thioacetate and hydrolysis).^80^ The resulting “thiol-subtilisin” could hydrolyse activated aryl substituted ester bonds. However, this cysteine variant lost its protease activity and was found to be 100-fold less active than the parental enzyme towards activated esters, despite the higher nucleophilicity of the free thiol.^81^,^82^ In another study, selenosubtilisin was created by converting the active site serine residue into selenocysteine.^57^,^58^,^83^ The selenium-containing protein was shown to be a reductase; alkyl peroxides could be converted to their alcohol equivalents under the action of this modified enzyme using thiophenol as a source of oxidant (Fig. 1a).^57^ The artificial enzyme exhibits reaction rates comparable to those of natural enzymes. While mechanistic insights are not available, the selenosubtilisin displays an inverted enantioselectivity in the kinetic resolution of racemic peroxides when compared to the native enzymes (Fig. 1b).^58^

**Fig. 2:**
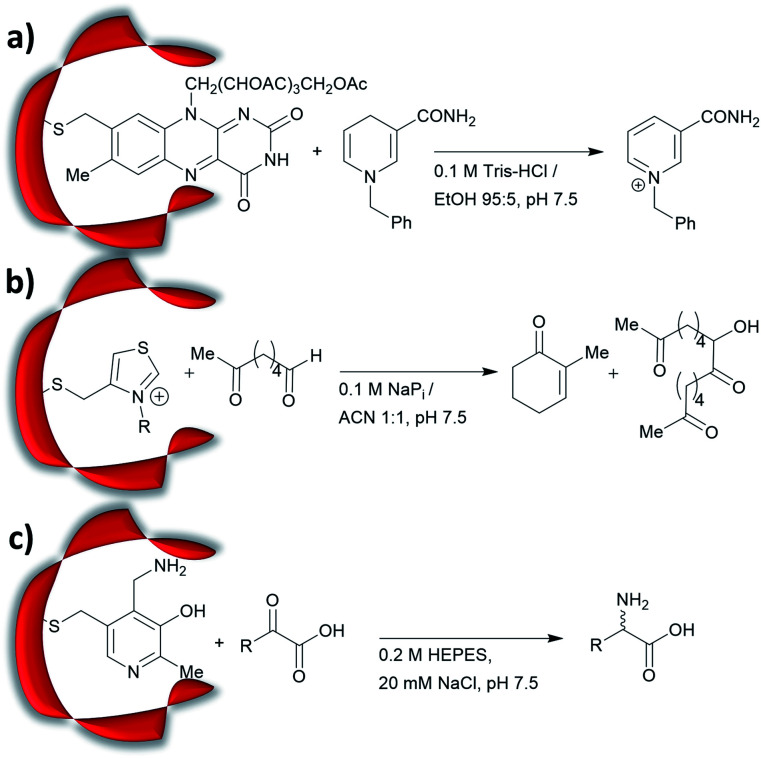
Artificial enzymes created by covalent modification of cofactors: (a) artificial flavopapain used for the oxidation of BNAH; (b) artificial thiazolopapains for C–C bond formation; and (c) artificial ALBP–pyridoxamine for enantioselective reductive amination. Tris = tris(hydroxymethyl)aminomethane, HEPES = (4-(2-hydroxyethyl)-1-piperazine-ethanesulfonic acid), ALBP = adipocyte lipid binding protein.

Cysteine, due to its nucleophilic nature, is most frequently modified with cofactors for the creation of new organocatalytic artificial enzymes. An artificial oxidoreductase was created by linking the catalytically active cysteine residue of the protease papain to flavins. Using oxygen for oxidation, the resulting “flavopapain” was able to oxidise NADH and its derivatives at a rate 50-fold higher than that by flavin alone (Fig. 2a).^84^ Similarly, the natural cofactor thiamine was introduced to papain. The resulting “thiazolopapain” was one of the early artificial enzymes that can mediate C–C bond formation.^59^ Nevertheless, activity was suboptimal, as the model cyclisation reaction of 6-oxo heptanal required six days to reach completion with a significant portion of substrate transformed in dimerisation byproduct (Fig. 2b). A third cofactor, pyridoxamine, was used to label the adipocyte lipid binding protein.^60^ The pyridoxamine protein complex could successfully mediate the production of a wide range of amino acids with modest to excellent enantioselectivity with enantiomeric excess (ee) up to 94% (Fig. 2c).

Recently, an alternative labelling strategy based on the metabolism of carbapenems by penicillin binding proteins was developed.^61^ In this work, secondary amine containing penicillin derivatives were anchored to beta-lactamase and the covalently modified protein was employed in a conjugate addition of nitromethane to cinnamaldehyde, giving moderate yields and low enantioselectivities (20–27%, e.r. ≈ 55 : 45).

These studies lay the foundations for the future development of protein-hosted organocatalysis.^57^,^84^,^85^ Chemical methodologies for protein labelling have vastly diversified and improved in recent years, showing fine-tuned reactivity and biocompatibility with labelling achieved within live cells.^86^–^94^ One can anticipate that efficient artificial enzymes can be made by adapting these novel technologies.

## *De novo* design/laboratory evolution

### Development of *de novo* enzymes

The increase in computational power and applicable software, including Rosetta and ORBIT,^95^ has accelerated the development of *de novo* enzyme design.^96^,^97^ The first stage of *de novo* enzyme design is the *in silico* generation of a “theozyme,” a theoretical arrangement of side chain residues and bioavailable molecules (water and ions) that can stabilise the rate-limiting transition state(s) of a chosen reaction.^98^ This assembly of theozyme is subsequently transformed into an experimentally tangible protein structure through evaluations based on calculated parameters (*e.g.* geometry and energy) by screening of available protein structures available in repositories.^49^,^95^ Eventually, the best options are recombinantly produced for characterisations. The initial *de novo* enzymes are typically inefficient and are not selective. Thus, laboratory evolution is used to enhance both catalytic activity and reaction profile. This pathway led to the formation of a highly competent and promiscuous *de novo* Kemp eliminase,^99^,^100^ retro-aldolases (RA)^40^,^43^,^44^,^63^,^65^–^68^ and Diels–Alderases.^52^,^101^ Here, we will focus on retro-aldolases which bear a catalytically active lysine for iminium and enamine catalysis.

**Fig. 3:**
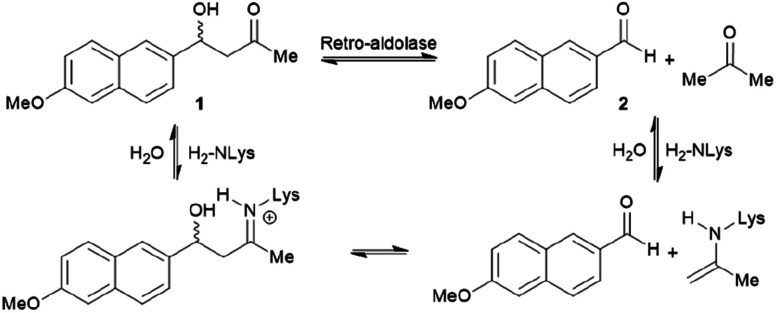
Net reaction of methodol (**1**) cleavage catalysed by the *de novo* designed retro-aldolases and important intermediates.

Retro-aldolases are a class of *de novo* designed enzymes capable of catalysing retro-aldol reactions *via* formation of an iminium intermediate.^68^ Retro-aldolases have been created from a theozyme that is able to mediate cleavage of the fluorogenic compound methodol (**1**, Fig. 3).^68^ The reaction was selected to allow for facile screening as the retro-aldol product naphtaldehyde (**2**) is fluorescent.

The most effective theozyme in terms of rate enhancement in the recombinantly produced protein contains a catalytically active lysine residue within a hydrophobic binding pocket and a strategically positioned water molecule that helps mediating formation of the Schiff base intermediate.^68^ Interestingly, this artificially designed network was found to be catalytically more active than those made based on naturally found proton shuffle networks. Computational tools, such as RosettaMatch,^96^ were recruited to dock the theozyme into a protein scaffold, creating a suitable host for the artificial active site.

**Fig. 4:**
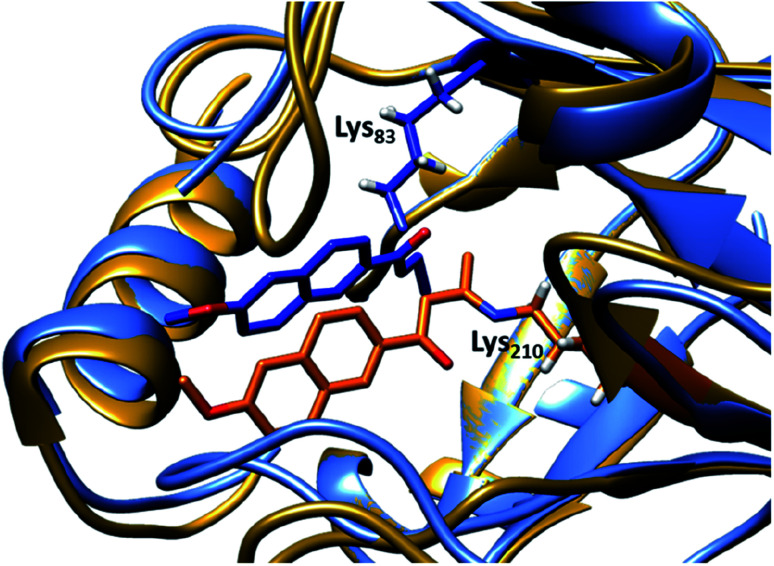
Overlay of crystal structures of the first retro-aldolase RA95.0 (gold & orange, PDB: 4A29), in which Lys210 is responsible for forming the Schiff base intermediate with a 1,3-diketone inhibitor and the evolved variant RA95.5-8 (light & dark blue, PDB: ; 5AN7) that carries a novel catalytically active Lys83 (in complex with a 1,3-diketone inhibitor).

Indole-3-glycerol phosphate synthase,^102^ a TIM-barrel protein fold, was identified for hosting the theozyme. Further adjustment of the residues surrounding the transition state was made using RosettaDesign, which among other purposes enables optimisation of residue interactions around the active site.^103^ Among these active models, the variant RA95.0 with the catalytically active lysine at position 210 (apparent p*K*_a_ = 8.1, Fig. 4) was identified as the most promising candidate. Experimentally, RA95.0 is able to mediate cleavage of methodol (**1**) with catalytic efficiency (*k*_cat_/*K*_M_) of ∼0.19 M^–1^ s^–1^ and selectivity for *S* over *R* (2.3 : 1).^66^,^67^ To create an enzyme with improved performance, regions at and around the active site of RA95.0 were subjected to iterative cassette mutagenesis, a form of saturation mutagenesis where pre-synthesised and mutated DNA strands are inserted into the gene by restriction enzyme digest and ligation.^104^ By combining mutations of the most active single variants, a highly improved variant RA95.5, which has six mutations in total, showed 73-fold increase in catalytic efficiency when compared to RA95.0 (*k*_cat_/*K*_M_ = 14 M^–1^ s^–1^ with 3 : 1 *R*-to-*S* selectivity). Crystallographic studies revealed that the T83K mutation in RA95.5 created a second reaction centre, in addition to Lys210, both capable of forming Schiff base intermediates. This finding indicated that the active site underwent restructuring, and further refinement was needed (see below).^66^,^67^ In particular, the replacement of T83K mutation shifted the p*K*_a_ of Lys210 to 7.6, to which the authors attributed the improved performance.

Additional laboratory evolution studies of the entire gene (error-prone PCR and DNA shuffling) created the variant RA95.5-5 that has an additional six mutations (compared to RA95.5) and demonstrated significantly improved activity (>20-fold, *k*_cat_/*K*_M_ = 320 M^–1^ s^–1^, and selectivity 5 : 1 *R* over *S*). Crystallographic studies illustrated that Lys83 transformed into the only reaction centre for the methodol (**1**) cleavage, indicating that there is a switch in location of the residue responsible for catalysis.^66^ Restructuring of the active site was likely unpredictable during the initial design, highlighting that randomness is a key element during the evolution of an efficient enzyme. Finally, a last three rounds of laboratory evolution yielded the variant RA95.5-8 (Fig. 4), which contains substitutions at both the active site and distal positions, and its catalytic efficiency (*k*_cat_/*K*_M_) was measured to be 850 M^–1^ s^–1^.^66^

### Aldolase evolution

Showcasing the power of ultra-high throughput screening methods, microfluidic fluorescence-activated droplet sorters (FADS) were used to further improve the performance of the retro-aldolase. The resulting variant RA95.5-8F displayed 13 mutations and a 30-fold higher activity (*k*_cat_/*K*_M_ = 34 000 M^–1^ s^–1^ for (*R*)-methodol (*R*-**1**) with 480 : 1 *R* over *S* selectivity).^64^ Such impressive improvement was attributed to the genesis of a catalytic Lys-Tyr-Asn-Tyr tetrad for proton shuffling. The tetrad forms a hydrogen bonding network which transfers proton to and from the reaction centre, stabilising formation of reaction transition states. RA95.5-8F was the first RA to be able to mediate aldol reactions between acetone and various aldehydes (*i.e.* an aldolase). It should be noted that previous RA's were inhibited by the formation of Schiff base with these aldehydes, whereas RA95.5-8F selectively forms enamines with acetone.

### Expanding the reaction profile of the RA95 family

**Fig. 5:**
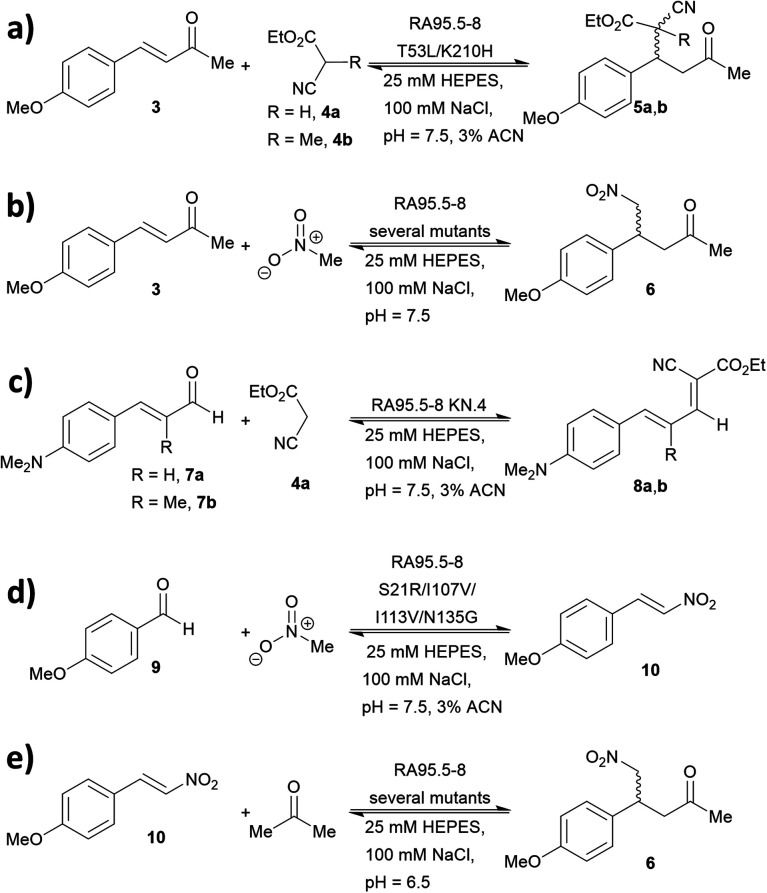
Promiscuity of RA95.5-8 and variants for carbon–carbon bond forming reactions. Iminium catalysis includes: (a) conjugate addition of carbon nucleophiles; (b) conjugate addition of nitromethane; (c) Knoevenagel condensations of carbon nucleophiles with α,β-unsaturated aromatic aldehydes and (d) Henry addition of nitromethane to aromatic aldehydes. Enamine catalysis includes: (e) conjugate addition of acetone to nitrostyrene.

A series of studies were conducted to expand the versatility of the RA95 family to catalyse different reactions (Fig. 5). Iminium catalysis mediated by RA95.5-8 was used as a means to mediate carbon–carbon bond formation, including conjugate additions (Fig. 5a and b),^40^,^42^,^44^ Knoevenagel (Fig. 5c)^43^ and Henry condensations (Fig. 5d).^41^ Enamine catalysis was also explored in the nitro-Michael addition of acetone to nitrostyrenes (Fig. 5e).^42^ In some cases, formation of reactive iminium species was verified by reduction of the intermediates followed by mass spectrometric analysis.^43^,^44^

During the course of optimising RA95.5 to mediate different transformation (Fig. 5), several notes have been learned. Firstly, there is a positive correlation between stereoselectivity and catalytic efficiency.^63^–^68^ Nevertheless, it should be noted that, during the optimisation process, stereoselectivity may be weak because the active site undergoes reconstruction (*e.g.* re-locating the catalytic residue);^40^,^41^,^44^ eventually, stereoselectivity resumes and variants with kinetic parameters and selectivity similar to those of natural enzymes can be achieved. Furthermore, refined artificial enzymes often possess properties similar to those of natural enzymes. For instance, loop flexibility and residues that are distant from the active site (secondary shell and protein surface) could greatly affect the performance of the catalysis.^41^,^42^,^44^,^105^,^106^ In another instance, it was indicated that catalysis is partially driven by a negative activation heat capacity, which is considered as a result of tight binding to the transition state forming an ordered complex.^62^ Finally, the computationally designed 248-residued RA can be modified at approximately 30 positions. This signifies the genetic “plasticity”^107^,^108^ of RA and echoes the fact that TIM-barrel fold is found in at least 15 families of enzymes.^109^–^111^ The coupling of computational design (rational) and laboratory evolution with high-throughput screening (randomness) has proven to be an effective approach to create *de novo* enzyme. In recent years, this technology has been combined with others, including genetic code expansion (see below). We anticipate that the family of *de novo* enzymes will soon be vastly expanded.

## N-Terminal proline

### 4-Oxalocrotonate tautomerase

**Fig. 6:**
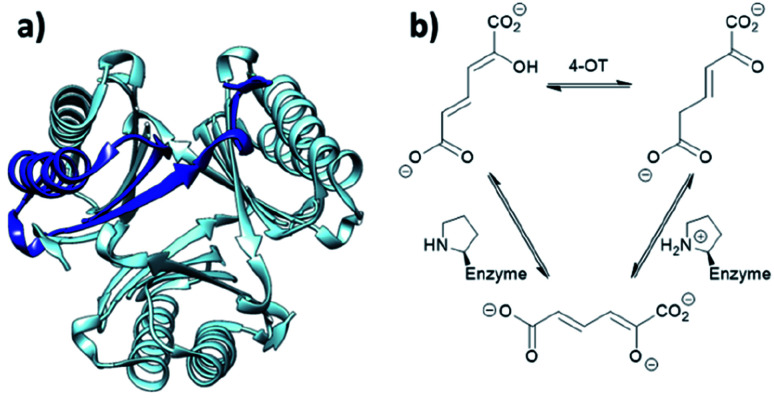
(a) Hexameric crystal structure of 4-OT (PDB: 4X19), monomer subunit highlighted in dark blue. (b) Native reaction of 4-OT, showing the net reaction in the upper part and the function of the N-terminal proline as general base below.

When located at the N-terminus of a protein, proline offers a secondary amine that can be used for iminium- and enamine-based organocatalysis. One such example is 4-oxalocrotonate tautomerase (4-OT) from *M. putida*, which is composed of six homologous monomers carrying a catalytic N-terminal proline (Fig. 6a).^112^ Naturally, this residue acts as a general base, catalysing the tautomerisation of a dienol into an unsaturated ketone (Fig. 6b).^38^,^113^,^114^ Interestingly, this proline residue forms iminium intermediates with various carbonyl substrates.

**Fig. 7:**
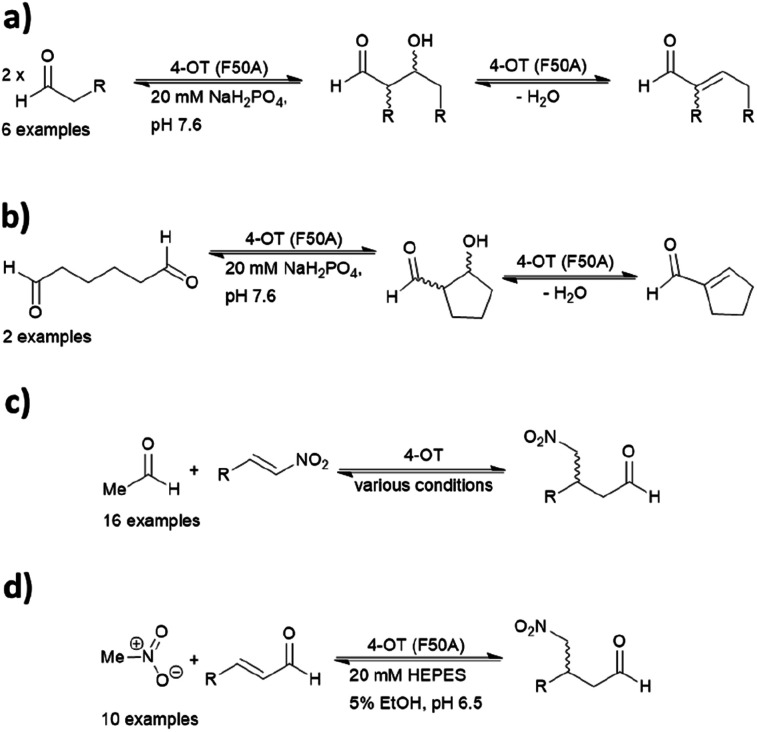
Reactions catalysed by 4-OT include (a) intermolecular aldol; (b) intramolecular aldol; (c) nitro-Michael addition; and (d) enamine catalysed conjugate addition. 4-OT = 4-oxalocrotonate tautomerase.

Because of its significant substrate promiscuity, 4-OT has been used as an organocatalyst for chemical transformations. It has been demonstrated that 4-OT is able to catalyse enamine-based aldol reactions (Fig. 7a and b)^37^,^70^ and conjugate additions (Fig. 7c).^71^ Additionally, 4-OT has been exploited for iminium catalysis, including the conjugate addition of nitromethane (Fig. 7d).^115^ Reduction of the intermediate iminium ion by sodium cyanoborohydride and subsequent mass spectrometry analysis provide evidence that supports the formation of the iminium intermediate.^73^

Mutagenesis *via* a combined computational and experimental approach has led to the identification of enhanced variants. Three residues in proximity were found to be crucial for catalysis, including Phe50, Met45 and Ala33. Mutability landscapes were used to determine ‘residue hotspots.’ The experiment consisted of singly mutating all amino acids with the exception of the catalytic Pro1.

Protein solubility of single point mutations was first assessed, followed by an activity screen of the tautomerization reaction and subsequently the Michael addition. An F50A mutation resulted in an increase of catalytic efficiency by a factor of 600 for cross-coupling aldol reactions.^72^ In contrast, when both Phe50 and Met45 were replaced with valine and tyrosine respectively, the resulting variant was more effective at self-condensation reactions. The F50V/M45Y double mutant resulted predominantly in the *R* product, whereas a third mutant A33D selectively yielded the *S* enantiomer in the conjugate additions of acetaldehyde to β-nitrostyrenes.^69^ Crystal structures of the two mutants have been obtained, but the N-terminal region was not resolved likely due to its inherent flexibility. Hence, the actual assembly in the active site remains unclear.

**Fig. 8:**
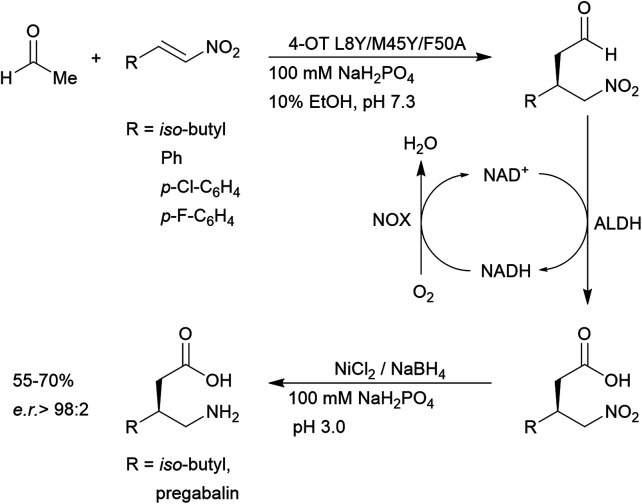
Chemoenzymatic synthesis of pregabalin and three of its analogues using 4-OT. NAD = nicotinamide adenine dinucleotide, ALDH = aldehyde dehydrogenase, NOX = NADH oxidase.

4-OT and its variants have been used for a range of applications including enzymatic^115^ and chemoenzymatic cascades,^116^ alongside whole cell catalytic systems.^117^–^119^ The anti-anxiety drug pregabalin and three of its analogues were synthesised by coupling the 4-OT reaction with catalysis by aldehyde dehydrogenase (ALDH, Fig. 8).

Acetaldehyde was added stereoselectively to α,β-unsaturated nitro substrates under the action of a 4-OT variant, followed by oxidation by ALDH to yield the corresponding carboxylic acid (Fig. 8).^116^ To recycle NADH, a cofactor recycling system operated by NAD oxidase (NOX) was included. Lastly, the nitro group was reduced to the amine using sodium borohydride in the presence of nickel chloride. These applications present evidence that protein-based organocatalysis can be used in combined synthesis which may not be readily achievable using traditional organocatalytic systems.

Utilising only natural residues with no chemical modification needed, the N-terminal proline approach is arguably the simplest in establishing a biocompatible organocatalytic system. As a range of reactions have already been established, 4-OT is an attractive system for performing organic reactions in biological contexts. However, a major limitation is that it is only able to catalyse secondary amine organocatalysis. Other useful organocatalytic transformations (based on *e.g.* thiourea or counterion based catalysis) are unavailable and thus other approaches must be employed.

## Genetic code expansion

### Fundamentals of genetic code expansion

**Fig. 9:**
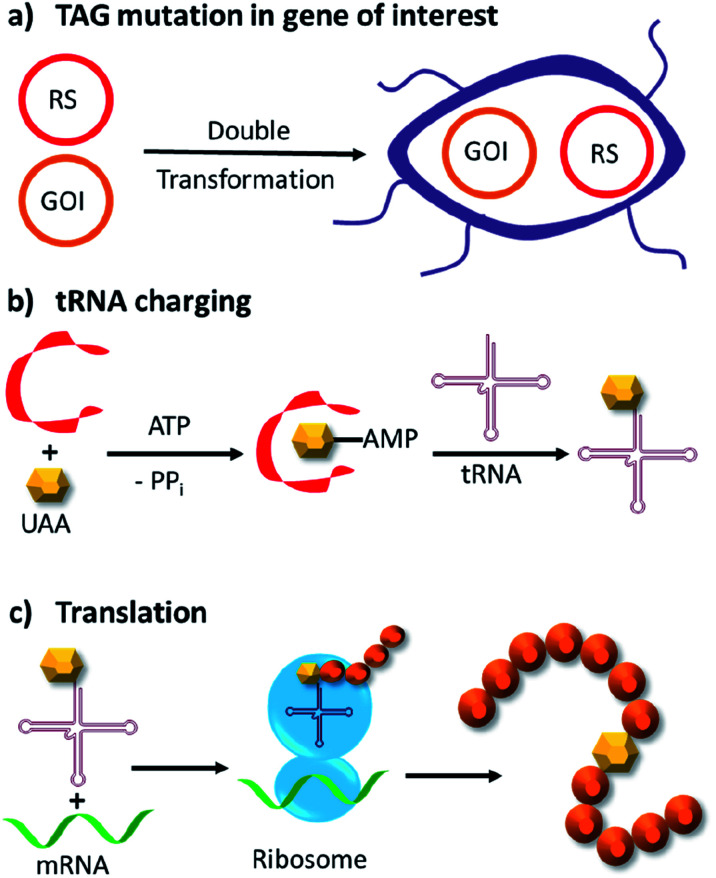
Incorporation of unnatural amino acids by genetic code expansion in *E. coli*. (a) Double transformation of two plasmids, in which one bears an exogenous amino acid tRNA synthetase (RS) and cognate tRNA, whereas the other contains the gene of interest (GOI) with a site-specific TAG mutation. (b) Expression of the tRNA synthetase and addition of the unnatural amino acid (UAA) allows the tRNA to be charged with the UAA. (c) Ribosomal translation of the GOI with the unnatural amino acid incorporated site specifically into the protein. ATP = adenosine triphosphate, PP_i_ = inorganic pyrophosphate.

Genetic code expansion enables site-specific incorporation of unnatural amino acids, which can be used to mediate bioorthogonal chemical reactions. To achieve this goal, a pair of orthogonal aminoacyl-tRNA synthetase/tRNA pair is needed. Specifically, the orthogonal tRNA decodes a blank codon, commonly the amber stop codon (TAG) as it is often the least used codons in most organisms. To produce recombinant proteins that contain unnatural amino acids in *E. coli*, pyrrolysyl-tRNA synthetase/tRNA and tyrosyl-tRNA synthetase/tRNA pairs from archaea are the most versatile and popular choices.^120^ The pyrrolysyl-tRNA synthetase/tRNA pair is particularly useful as it naturally decodes the amber codon.^121^ To incorporate the unnatural amino acid, a TAG codon is then introduced into the gene of interest at the position of choice. Double transformation of *E. coli* with plasmids containing the gene of interest and the synthetase are conducted. By including the unnatural amino acid in the medium, the orthogonal synthetase specifically charges the orthogonal tRNA with the unnatural amino acid, which will allow for production of full-length protein with the unnatural amino acid at the desired position. To date, over 200 unnatural amino acids can be genetically incorporated into a protein of interest using this technique, so there exists a vast opportunity to exploit these unnatural amino acids for organocatalytic transformations (Fig. 9).^91^

### The multidrug regulator protein LmrR

**Fig. 10:**
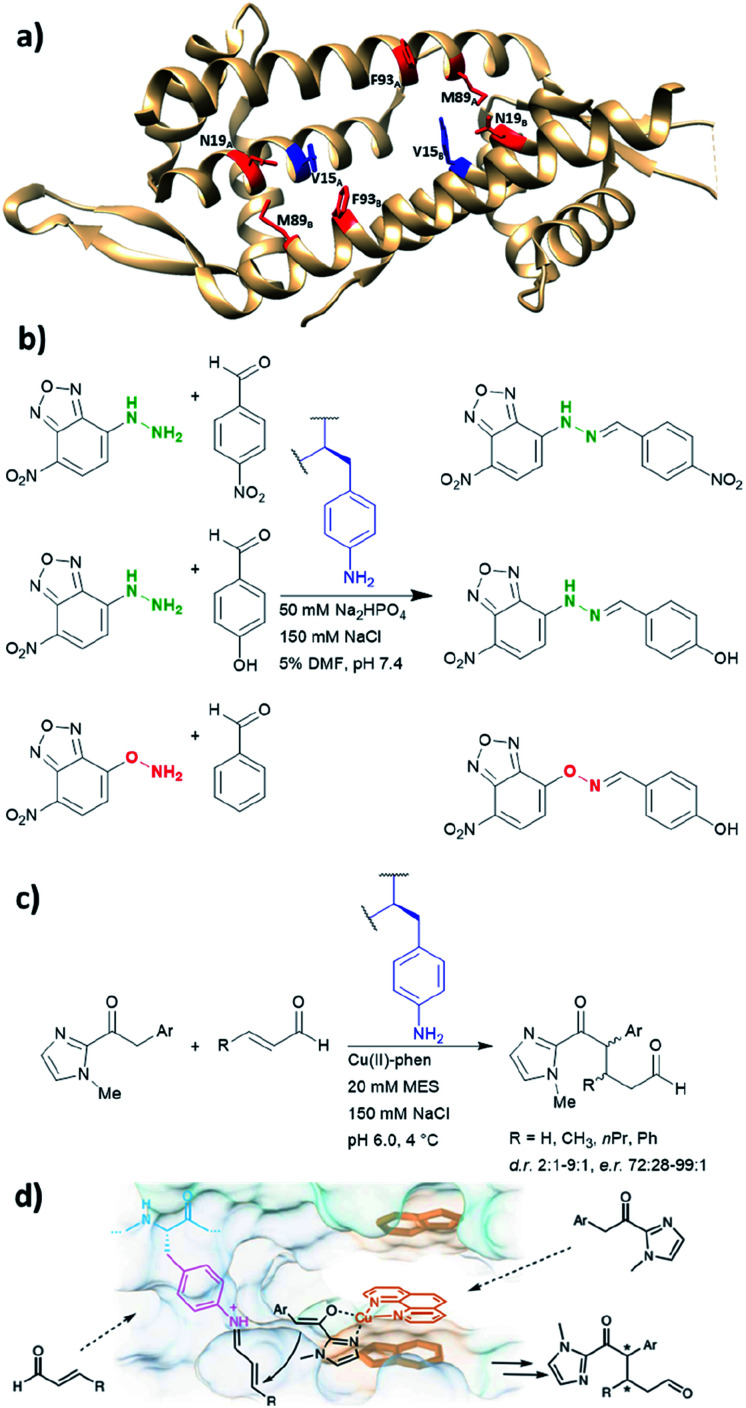
(a) Crystal structure of the homodimeric protein LmrR (PDB: 3F8F). Residues in red are those targeted for unnatural amino acid incorporation (Asn19, Met89, Phe93). Residues in blue (Val15) show the most promising result, when they are replaced with the unnatural amino acid *p*-amino-phenylalanine. (b) Hydrazone and oxime ligation performed by the unnatural amino acid. (c) Conjugate addition catalysed by dual substrate activation using LmrR V15pAF and a copper complex. (d) Postulated mechanism for the conjugate addition *via* dual activation from (c). (d) Adapted from ref. 122, ; https://www.nature.com/articles/s41929-019-0420-6, with permission of Springer Nature, copyright 2020. Further permissions related to the material excerpted should be directed to Springer Nature. DMF = dimethylformamide, phen = phenanthroline, pAF = *p*-aminophenylalanine.

LmrR is a dimeric protein isolated from *Lactococcus lactis* that has a hydrophobic pore in the centre, allowing for the recruitment of organic molecules (Fig. 10a). Four positions located within the hydrophobic pore (Val15, Asn19, Met89 and Phe93) were individually mutated to a TAG codon and tested for the incorporation of the unnatural amino acid *p*-azidophenylalanine under the action of an evolved tyrosyl-tRNA synthetase from *Methanococcus jannaschii*. The azido group was chosen and subsequently reduced to the catalytically active aniline, because direct incorporation of *p*-aminophenylalanine proved to be challenging.^75^ The designer enzyme was then tested for hydrazone and oxime formation. It was found that unnatural amino acid replacement at the Val15 position yielded the most promising result (Fig. 10b).^75^ Laboratory evolution was used to screen the library variants in 96 well plates by measuring the loss of the UV absorbance from the substrate.^50^ The resulting variant which carries additional mutations, including A11L, N19M, A92R and F93H, showed a 74-fold increase in catalytic efficiency. Based on the knowledge of these positions from previous structures, Leu11 and Met19 are thought to help position the aniline in a more “reaction-ready” position. Furthermore, Arg92 was reasoned to stabilise the build-up of negative charge that appears during the condensation of the aniline with the carbonyl group. Lastly, His93 was proposed to serve as proton shuttle assisting in the formation of iminium ion intermediates and promoting the transamination processes.

Recently, the *p*-aminophenylalanine/LmrR system has been further modified for a novel dual substrate activation strategy.^122^ Through combination with a supramolecularly bound Lewis acidic Cu(ii) complex, the resulting artificial enzyme was able to mediate a Michael reaction that involves both formation of a Cu-enolate and an organocatalytic iminium intermediate. Yields of this novel reaction mode were up to 90%, with d.r. and ee up to 9 : 1 and >99% respectively. This work highlights that importance of developing different approaches to artificial enzyme design (*e.g.* genetic code expansion and supramolecular approach), as proteins can be used to host multiple catalytic centres for coupled reaction cascades.

### *De novo* designed BH32

**Fig. 11:**
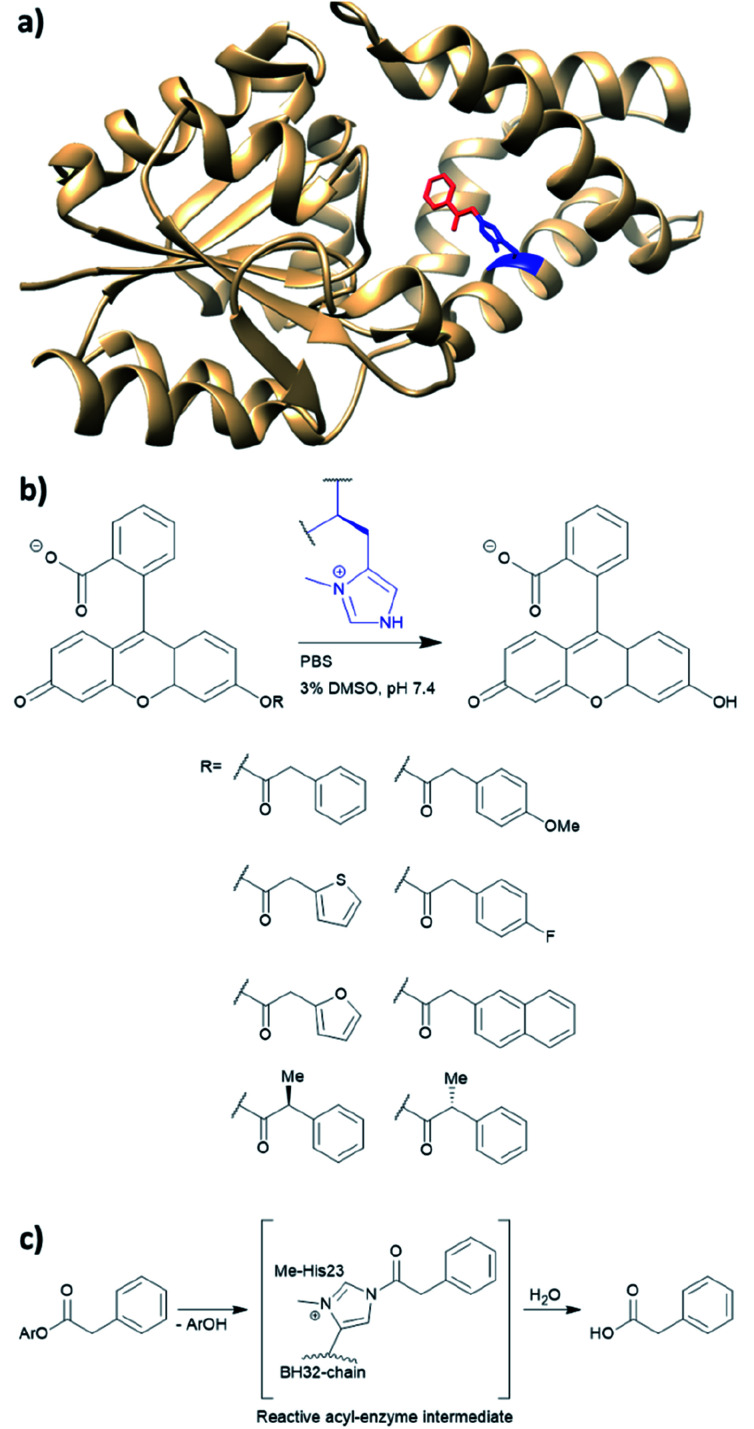
(a) Crystal structure of the protein BH32 (PDB: 6Q7N). Residue in blue is the unnatural amino acid methyl-histidine bound to acetophenone (red). (b) Substrate scope of the ester hydrolysis performed by BH32 with the unnatural amino acid Me-His. (c) Shortened mechanism of the acyl enzyme intermediate formation of BH32 Me-His23 with aromatic esters. PBS = phosphate-buffered saline, DMSO = dimethyl sulfoxide.

BH32 is an enzyme originally created by Rosetta to perform the Morita–Baylis–Hillman reaction,^101^ and this protein has been further re-engineered into a potent hydrolase through the combined use of genetic code expansion and laboratory evolution.^74^ Substitution of the catalytic His23 with methyl-histidine was achieved by using an evolved variant of the pyrrolysyl-tRNA synthetase and its cognate tRNA (Fig. 11a). The resulting enzyme was able to perform ester hydrolysis for a range of compounds that fluoresce upon reaction (Fig. 11b). Screening for variants with improved activity was performed using 96 well plates on a plate reader where formation of the fluorescein product could be monitored. Six mutations resulted in a 15-fold increase in enzyme activity. Mutations resulting from the evolution were L10P, A19H, S22M, E46N, P63G and D125G. Based on the data derived from crystallography and kinetic investigations, the authors concluded that the aromatic ester formed between the substrate and Me-His was significantly more prone to hydrolysis (Fig. 11c). In contrast, the neutral acyl enzyme intermediate formed from the natural amino acid histidine hydrolyses slowly under the same condition.

The technique of genetic code expansion allows exploration beyond the limit of what natural amino acids offer, thus holding great promise in contemporary enzymology. Incorporation of unnatural amino acids *in vivo* enables laboratory evolution in a fashion similar to those of natural enzymes. Consequently, artificial enzymes made by this fashion can also be applied to whole cell catalysis or synthetic biological pathways. However, the efficiency of incorporation greatly depends on the unnatural amino acid used. The choice of protein to harbour the amino acid also needs to be considered carefully. Both LmrR and BH32 have been previously used in artificial enzyme design (LmrR for artificial metallo-enzymes and BH32 was computationally designed for carbon–carbon bond forming reactions).^47^,^92^ Both examples have shown promise in performing biocompatible organocatalysis. As genetic code expansion has become more readily available, this technique will likely gain increasing popularity in the future of enzyme design.

## Non-covalent supramolecular systems

### Harnessing non-covalent interactions

**Fig. 12:**
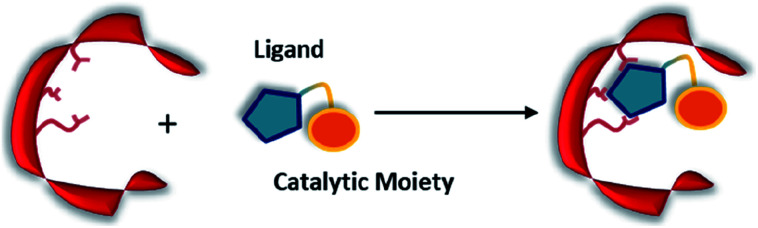
Combination of a catalytically inactive protein scaffold with a ligand–catalyst conjugate leads to a catalytically active supramolecularly assembled protein complex.

Non-covalent but strong protein–ligand interactions have been exploited to generate organocatalytic artificial enzymes. In these systems, a catalytic moiety is covalently attached to a section of a ligand that is only weakly involved in protein binding and introduced to the protein partner. Consequently, the resulting protein–ligand complex is converted into a potential catalytic entity (Fig. 12).

When compared to other approaches, a supramolecular complex has little restrictions on the choice of the catalytic motifs. Preparation of the modified ligands tends to be straightforward, involving simple synthetic techniques such as amide bond coupling and click chemistry.^35^,^39^,^123^–^126^ Hence, the supramolecular approach enables researchers to screen activity for a broad range of candidates within a short period of time. In addition, the protein hosts can still be engineered *via* rational design or laboratory evolution.^46^,^48^,^127^–^129^ To this end, the supramolecular approach is an important technique for creating artificial enzymes. As a rule of thumb, the supramolecular catalytic complexes are built based on protein–ligand interactions that have dissociation constants (*K*_D_) ranging from low μM to pM.^123^–^125^,^130^

The ligand needs to possess a site for easy functionalisation while causing minimal effect on protein–ligand interaction. One such pair is the (strept)avidin and biotin, whose *K*_D_ value is approximately 10^–14^ M^–1^.^130^ The (strept)avidin–biotin system has already been exploited in the late 1970s to tether a rhodium catalyst to the valeric motif of biotin for asymmetric hydrogenations.^131^ Subsequently, a variety of streptavidin based artificial metallo-enzymes operated by iridium, rhodium, ruthenium and palladium have been reported.^35^,^48^,^123^,^126^,^127^,^132^,^133^ Below we describe two different types of organocatalytic artificial enzymes based on biotin–streptavidin.

**Fig. 13:**
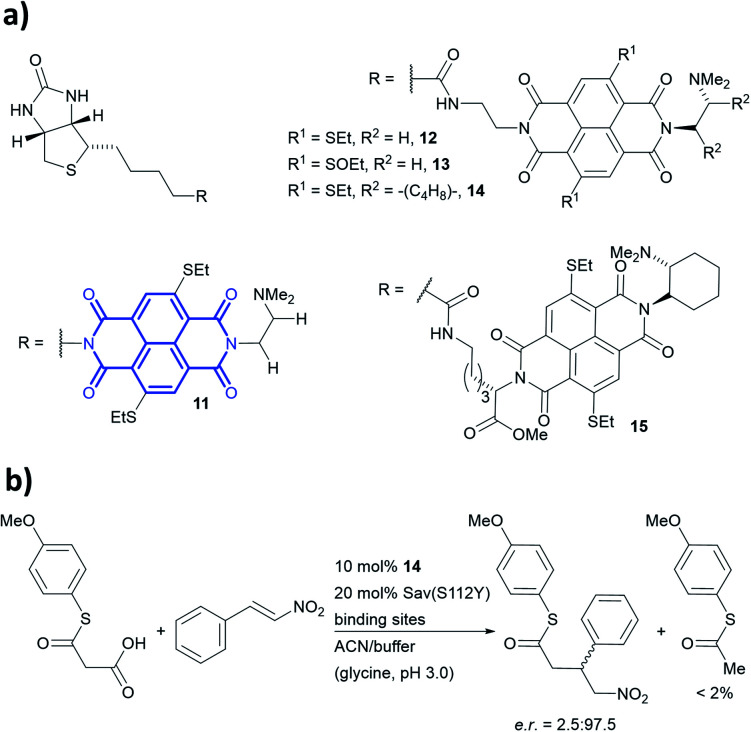
(a) Reported biotin–NDI–amine organocatalyst conjugates, NDI fragment in bold and blue.^36^ (b) Decarboxylative Michael addition reaction catalysed by **14** within S112Y mutant streptavidin. Sav = streptavidin.

Anion–π-catalysis has become a contemporary topic in organocatalysis.^9^,^134^–^139^ In this catalytic mode, anion intermediates formed during the reaction can be stabilised by π-acidic molecules such as naphthalenediimides (NDIs, bold blue core in **11**, Fig. 13a), which possess a positive quadrupole moment. This consequently facilitates organic transformations such as conjugate additions (Fig. 13b). Whereas all natural aromatic amino acids are π-basic and interact with cations, the streptavidin–biotin technology was recently used to create an organocatalytic artificial enzyme that drives catalysis by anion–π interactions.^36^,^76^

To add anion–π interactions into the repertoire of enzyme catalysis, a combined chemical and genetic screening approach was used. A library of five bifunctional catalytic moieties were attached to biotin (compounds **11–15**, Fig. 13a) that contain both an NDI motif and a tertiary amine connected through a linker of different length. The π-acidic surface of the NDI motif was proposed to be able stabilise the enolate intermediate formed in the reaction, whereas the tertiary amine acts as a base and localises the enolate intermediate over the NDI moiety.^140^ Hence, their ability to mediate a decarboxylative alkylation between thioester malonates and nitrostyrenes was evaluated (Fig. 13b).^36^

Ligand **14** was identified to be most reactive, and the activity was screened using a streptavidin library of 20 variants. The combination of ligand **14** and S112Y variant yields an organocatalytic artificial enzyme that selects for product formation over the decarboxylated starting material at a ratio >30 : 1. The conversion in ACN : glycine buffer at pH 3.0 was found to be 90% with e.r. up to 97.5 : 2.5.

**Fig. 14:**
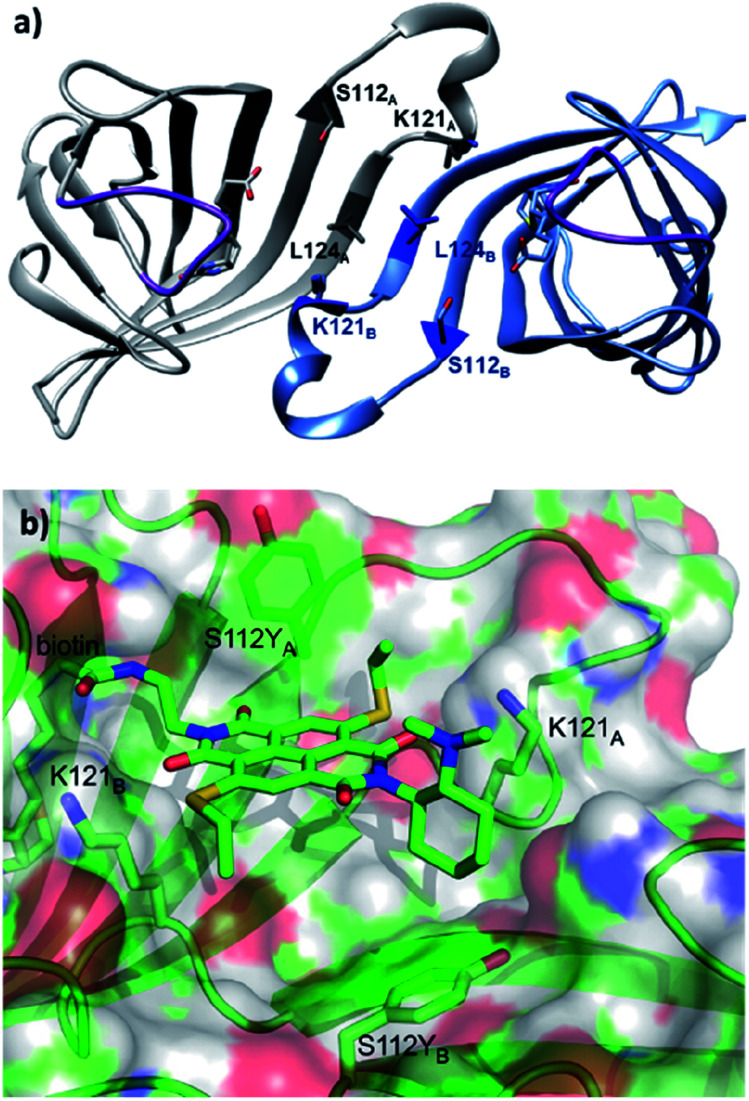
(a) Cartoon overview of the assembly of streptavidin with full binding-site occupation with d-biotin in both binding sites, monomeric unit in light blue. The *C*_2_ symmetric interface of two streptavidin subunits is illustrated (PDB: ; 1MK5). Amino acids Ser112, Lys121, and Leu124 are highlighted. Purple loop regions represent amino acids 46–52. The other two subunits of the homotetrameric streptavidin are omitted for illustration purpose. (b) Cartoon overview of a single binding-site of anion–π catalyst **14** in Sav S112Y. (b) Adapted from ref. 36, ; https://pubs.acs.org/doi/abs/10.1021/acscentsci.6b00097, with permission of ACS, copyright 2016. Further permissions related to the material excerpted should be directed to the ACS.

Based on the site-directed mutagenesis studies and docking simulations, a plausible mechanism operated by ligand **14**/Sav-S112Y was proposed. A medium sized linker between biotin and NDI (*i.e.* ligand **14**) is essential to accommodate the catalytic unit close to the biotin-binding vestibule, whilst not causing any steric clash. Large electron-withdrawing substituents at the NDI motif were found to weaken the binding (**13** *vs.* **14**, Fig. 13a), while a flexible dimethylene bridge instead of a rigid one (**12** *vs.* **14**, Fig. 13a) hampers both the conversion and selectivity. The tertiary amine/NDI motif locates in close proximity to the intersubunit interface of the homotetrameric streptavidin, which has a *C*_2_ symmetry (Fig. 14a). Hence, residues from both monomers can interact with the catalyst and substrates,^141^ and the docking studies revealed that the S112Y mutation from each monomer, namely S112Y_A_ and S112Y_B_, is essential to the catalysis by ligand **14**.

When the ligand is bound to monomer A, the NDI motif forms π–π-interactions with S112Y_A_; this is supported by the observation that the mutant S112E and S112W resulted in decreased activity, while the S112F mutant showed similar reactivity to that of S112Y (Fig. 14b). In contrast, S112Y_B_ shielded its own biotin binding site. Accordingly, optimal activity was obtained when the catalyst to free binding-sites ratio was kept at 1 : 2. The wild type lysine residues Lys121_A_ and Lys121_B_ anchor the NDI at the designated location. Furthermore, Lys121_A_ helped maintaining a low p*K*_a_ value for the tertiary amine of **14**, keeping it in its deprotonated form for reaction (even at pH 3.0). Mutation of Lys121 led to a detrimental effect on the activity and selectivity. This study revealed the intricate interactions between the residues and catalytic motif, thereby highlighting that screening of both ligands and variants is critical to obtain an efficient and selective supramolecular system.

**Fig. 15:**
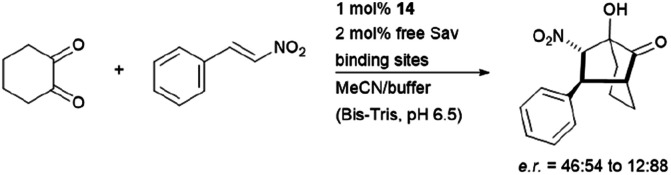
Domino-Michael–Aldol-reaction catalysed by **14**. Bis-Tris = bis(2-hydroxyethyl)amino-tris(hydroxymethyl)methane.

The hybrid catalyst system of streptavidin and conjugate **14** was further employed to perform a bioorthogonal domino-Michael–aldol reactions between diketones and nitrostyrenes (Fig. 15).^76^ With 1–5 mol% catalyst loading, the bicyclic products were obtained in moderate yields (≈50%), decent enantioselectivities (0–80% ee) and significant diastereoselectivity (>20 : 1) after screening with four streptavidin mutants. Interestingly, the protein–ligand assembly lead to an inversion of stereoselectivity when compared to the nascent biotin-catalyst conjugate.

The biotin-binding-sites in wild-type Sav are rather shallow, exposing a good portion of the catalytic moiety to the solvent. The lack of amino acid side chains in proximity makes mutational optimisation difficult to achieve (Fig. 14b). This led to the development of chimeric Sav variants, which contain insertions of amino acid loops around the biotin-binding sites of Sav like naturally occurring random loops or α-helices.^55^ Eight chimeric Sav variants containing random coils and alpha helix motifs inserted between residues 46–52 (purple region, Fig. 14a) and one with an addition at the C-terminus have been tested as host for the decarboxylative alkylation catalysed by ligand **14** (Fig. 13b). Though initially thought to increase stereoselectivity and reactivity, three of these chimeric protein hosts were completely inactive and the rest showed lower yields and enantioselectivities than the previously optimised mutant S112Y. Nonetheless, there are similar levels of selectivity for product formation over the decarboxylated starting material (>30 : 1).

### Secondary amine organocatalysis

**Fig. 16:**
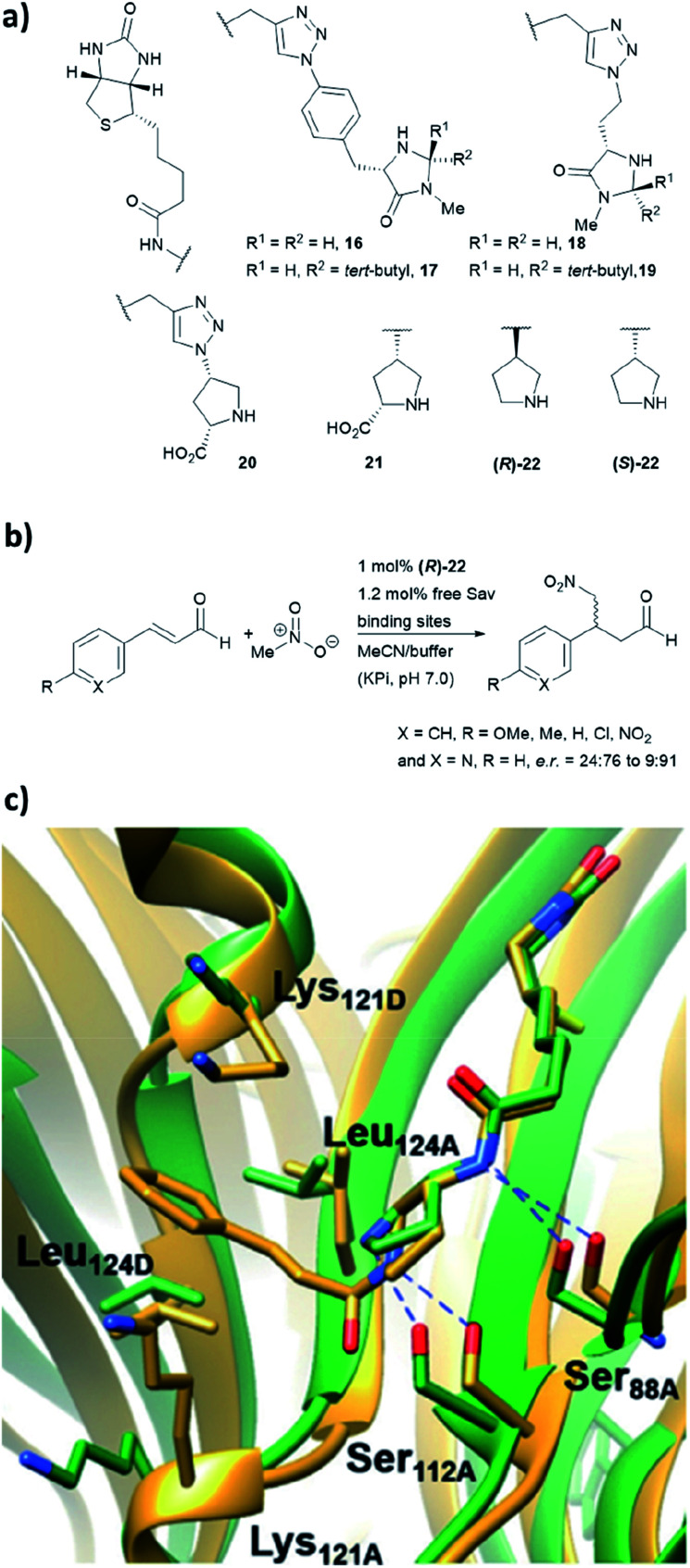
(a) Reported biotin–secondary amine organocatalyst conjugates.^39^ (b) Set of Michael addition reactions catalysed by **22** within a recombinant core streptavidin. (c) Cartoon overview of Sav:(*R*)-**22** crystal structure (green, PDB: ; 6GH7) and aminol adduct obtained from QM/MM simulations (golden), and it was adapted from ref. 39, which is an open access article under the terms of the Creative Commons Attribution License, published by Wiley-VCH, copyright 2018, the authors.

We have recently employed the streptavidin–biotin technology to create protein-based secondary amine organocatalytic systems. Seven biotinylated secondary amines (ligands **16–22**, Fig. 16a) were prepared *via* either copper-catalysed azide–alkyne cycloaddition or amide bond coupling reactions.^39^ These catalysts can be broadly segregated into three types: MacMillan-like imidazolidinones (**16–19**), prolines (**20**, **21**) and pyrrolidines (**22**, **23**), and their ability to catalyse the Michael addition of nitromethane to aromatic α,β-unsaturated aldehydes was tested (Fig. 16b).

Both (*R*)- and (*S*)-**22** alone are not enantioselective. However, when introduced to the tetrameric streptavidin, they were found to be able to mediate the model reaction with high reactivity and stereoselectivity.^39^ Moderate to good yields (37–80%) were obtained using only 1 mol% of protein catalyst and 1 : 1 MeOH/potassium phosphate buffer as reaction medium. Notably, these two protein complexes, namely Sav:(*R*)-**22** and Sav:(*S*)-**22**, differed by only one chiral centre, but their stereoselectivity was opposite, with the former favouring for the *S* enantiomer and latter for the *R* enantiomer. Through crystallographic and computational structural studies, the position of the secondary amine motif was found to be in proximity to Ser112_A_. The lysine and leucine residues located at the dimer interface (Lys121_A/B_ and Leu124_A/B_) dictate the face for which the intermediate was exposed for nucleophilic addition, thereby dictating both regio- (1,2 *vs.* 1,4-addition) and enantioselectivity (*R* and *S*) of the reaction (Fig. 16c).^39^

Based on the precedence of metallo-enzyme development and supramolecular capsules,^142^,^143^ two organocatalytic artificial enzymes that operate distinctively different mechanisms have been designed. Nevertheless, the conditions developed so far are not completely biocompatible, as acidic conditions (pH 3.0) and/or a large volume of organic co-solvents are needed (though the latter was mostly due to the use of substrate with poor solubility in aqueous environments).^23^ Furthermore, activity of these non-covalent complexes could be potentially optimised *via* laboratory evolution, as demonstrated by the existing Sav-based catalytic systems.^46^,^48^,^127^,^128^ Though being a robust and reliable system, issues related to the *C*_2_ symmetry of the intersubunit interface of Sav was only recently addressed by the creation of “dimeric” Sav.^141^ This “dimeric” Sav variant will facilitate mechanistic studies and the design of tailored and asymmetric scaffolds for chemical catalysis. In addition to the streptavidin:biotin system, other protein–ligand systems should serve as inspiration for novel protein-based organocatalytic systems, including the siderophore binding proteins^124^ or coumarin binding albumins.^144^

## Conclusions and outlook

In this review, we summarised five approaches which are currently employed to perform organocatalysis within proteins (Table 1). The supramolecular tethering and N-terminal proline approaches have proven to be successful, and chemical catalysis with improved biocompatibility has been exploited in different applications including chemo-enzymatic synthesis^116^ and gene switches.^145^ In turn, covalent modification, computational *de novo* design and genetic code expansion are anticipated to excel, as related technologies have vastly improved and gained popularity in recent years.^146^–^148^ Importantly, a much-improved system can be achieved by combining different approaches. This can be exemplified by the recent development of LmrR modified with dual catalytic groups.^122^ Genetic code expansion can also be used to introduce novel catalytic functionalities into a *de novo* designed enzyme active site. This has been demonstrated in a recently reported artificial metalloenzyme, in which the designed active site includes the unnatural amino acid bipyridylalanine for metal binding.^149^

Despite all these exciting opportunities, there are aspects that need to be immediately addressed in the area of organocatalytic artificial enzyme design. Notably, many of the current systems suffer from poor reactivity, with enzyme loadings up to 20 mol% needed for reaction. However, the development of RA95, aniline/LmrR and methylated histidine/BH32 have demonstrated that laboratory evolution is a feasible approach for activity improvement. The choice of protein scaffold and screening system likely play critical roles during the design. Furthermore, most protein-based organocatalytic systems are based on enamine and iminium catalysis that have similar counterparts in Nature. Useful bioorthogonal reactions that are frequently used in small molecule synthesis have not been tested, including α-fluorinations, aziridinations and Diels–Alder reactions.^4^ Also, sophisticated catalytic modes such as singly occupied molecular orbital (SOMO)^150^ activation or photo-radical chemistry^151^ can also be explored. In turn, other useful catalysts, including hydrogen bonding activators (thioureas and squaramides),^152^,^153^ N-heterocyclic carbene^32^,^154^ and ion pairing catalysis^155^ have yet been explored. Additionally, repurposing flavin-dependent enzymes for novel photo-redox reactions represent a valuable avenue for artificial enzyme design.^156^–^158^ In summary, this review illustrated that the design of artificial organocatalytic enzymes has become an exciting area of research and it will play critical roles in both chemical and synthetic biology research in future.

## Conflicts of interest

There are no conflicts to declare.
